# Algal Blooms and Cyanotoxins in Jordan Lake, North Carolina

**DOI:** 10.3390/toxins10020092

**Published:** 2018-02-24

**Authors:** Daniel Wiltsie, Astrid Schnetzer, Jason Green, Mark Vander Borgh, Elizabeth Fensin

**Affiliations:** 1 Department of Marine, Earth and Atmospheric Sciences, North Carolina State University, Raleigh, NC 27695, USA; dpwiltsi@ncsu.edu; 2 North Carolina Department of Environmental Quality, Division of Water Resources, Raleigh, NC 27699, USA; jason.green@ncdenr.gov (J.G.); mark.vanderborgh@ncdenr.gov (M.V.B.); elizabeth.fensin@ncdenr.gov (E.F.)

**Keywords:** freshwater blooms, cyanobacteria, cyanotoxins, microcystin, anatoxin-a, BMAA, North Carolina, SPATT, water quality

## Abstract

The eutrophication of waterways has led to a rise in cyanobacterial, harmful algal blooms (CyanoHABs) worldwide. The deterioration of water quality due to excess algal biomass in lakes has been well documented (e.g., water clarity, hypoxic conditions), but health risks associated with cyanotoxins remain largely unexplored in the absence of toxin information. This study is the first to document the presence of dissolved microcystin, anatoxin-a, cylindrospermopsin, and β-*N*-methylamino-l-alanine in Jordan Lake, a major drinking water reservoir in North Carolina. Saxitoxin presence was not confirmed. Multiple toxins were detected at 86% of the tested sites and during 44% of the sampling events between 2014 and 2016. Although concentrations were low, continued exposure of organisms to multiple toxins raises some concerns. A combination of discrete sampling and in-situ tracking (Solid Phase Adsorption Toxin Tracking [SPATT]) revealed that microcystin and anatoxin were the most pervasive year-round. Between 2011 and 2016, summer and fall blooms were dominated by the same cyanobacterial genera, all of which are suggested producers of single or multiple cyanotoxins. The study’s findings provide further evidence of the ubiquitous nature of cyanotoxins, and the challenges involved in linking CyanoHAB dynamics to specific environmental forcing factors are discussed.

## 1. Introduction

The eutrophication of waterways causes water quality issues worldwide and these may intensify with climate change [1,2,3,4]. One issue, linked to excess nutrient input from agricultural land and urbanized areas, is Harmful Algal Blooms (HABs) [5,6,7]. In freshwater systems and estuaries, HABs are typically dominated by cyanobacteria (or blue-green algae, CyanoHABs) [6,8,9] that can have multiple adverse effects on aquatic ecosystems, from the blocking of sunlight to benthic vegetation, to oxygen depletion that may kill fish [1,10,11,12]. Global annual estimates of the socioeconomic costs of CyanoHABs are significant and range from millions to billions of dollars (e.g., water monitoring and testing, drinking water treatment, adverse impacts on recreational use and fisheries) [13,14,15].

Various environmental factors impact the initiation, peak and demise of a CyanoHAB. Increased nutrients, mainly nitrogen (N) and phosphorus (P), have long been associated with bloom development [16,17], while other triggers, such as shifts in nutrient ratios throughout a bloom’s lifecycle, may also play a role in cyanobacterial composition shifts. Decreasing N:P ratios can promote bloom-forming cyanobacterial genera, capable of *N*-fixation [17,18,19,20,21], and low-flow conditions within lakes or estuaries reportedly favor the growth of cyanobacteria over other algal taxa [22,23,24]. Rising water temperatures have been linked to increased bloom activity [25,26,27] and to potential shifts from non-toxic to toxic strains [28]. At least a dozen cyanobacterial genera have been implicated with toxin production, and at least eight toxin groups have been characterized, of which microcystin (MCY) has been studied most extensively [13,29]. However, not all species within a genus can produce toxins and those that can, do not do so continuously. Field and laboratory studies show that MCY concentrations tend to be positively correlated with dissolved inorganic nutrients (mainly N and P), temperature and light levels [30,31,32]. For instance, temperature optima for MCY production by *Microcystis* and *Dolichospermum* strains were reported between 18 to 25 °C, and for *Dolichospermum* spp., temperature seems to influence which MCY congener is produced [32,33]. While many studies report that absolute nutrient concentrations are linked to cyanotoxin presence, shortages of certain nutrients that lead to shifts in nutrient ratios may be a factor. N-limitation was linked to increased MCY and anatoxin-a (ANA) concentrations by N-fixing members of the genera *Aphanizomenon*, *Cylindrospermopsis* and *Dolichospermum* [30,32], while P-limitation was associated with low MCY production by *Dolichospermum* spp. and *Microcystis* spp., and with low ANA levels in *Aphanizomenon* spp. [30,32]. In laboratory experiments, light levels of <20 µmol photons m^−2^ s^−1^ seem to be conducive to MCY production [30,32,34]. Overall, a better understanding of the complex interplay between environmental factors, cyanobacterial growth and/or the onset of toxin production is needed in order to mitigate, and ultimately, prevent, CyanoHAB-related issues within given environments.

Cyanotoxin consumption can harm fish, livestock, pets and humans in varying ways [35,36]. Exposure to MCY and cylindrospermopsin (CYN) can impair liver function and at high doses can be lethal [35,37,38,39]. ANA and saxitoxin (STX) are both neurotoxins [29,39]. ANA causes an overstimulation in neuromuscular junctions, leading to respiratory failure [39]. STX is responsible for paralytic shellfish poisoning (PSP), a condition that can cause paralysis and death in humans [40,41,42,43]. More recently, β-*N*-methylamino-l-alanine (BMAA) has been investigated for its connection to neurological diseases, including amyotrophic lateral sclerosis (ALS), Alzheimer’s disease and Parkinson’s disease [44,45,46]. While an increasing number of studies are addressing potential health risks due to these substances, major knowledge gaps remain in regard to exposure pathways, concentrations in the field and environmental triggers for toxin production.

A recent US-wide survey of over 1100 lakes showed that MCY was present in 32% of the tested lakes (range = below detection (BD) to 230 µg L^−1^; average = 3.0 µg L^−1^), and at least one of the cyanotoxins (MCY, CYN, STX or ANA) could be detected in 92% of the States [47]. CYN was reported in 4.0% (range = BD to 4.4 µg L^−1^; average = 0.6 µg L^−1^), STX in 7.7% (range = BD to 0.38 µg L^−1^; average = 0.06 µg L^−1^) and ANA in approximately 0.3% of samples (range and average not given) [47]. Comprehensive toxin surveys, especially for multiple toxins, are still rare, and risk assessment by the World Health Organization (WHO) is mainly based on chlorophyll *a* (Chl *a*) levels and cyanobacterial abundance ranges. As Loftin et al. [47] demonstrate, these metrics, in contrast to actual toxin information, can lead to an overestimation of MCY risk. This overestimation is partly due to the fact that, as aforementioned, not all cyanobacteria are toxin producers, and known toxic species do not produce toxins continuously. Under the Drinking Water Protection Act, the US Environmental Protection Agency (EPA) released national 10-day health advisories for MCY of 0.3 µg L^−1^ for infants and 1.6 µg L^−1^ for adults, and for CYN, 0.7 µg L^−1^ for infants and 3 µg L^−1^ for adults, based on body weight and water intake [48,49]. In December 2016, the EPA also suggested recreational guidelines of 4 µg L^−1^ for MCY and 8 µg L^−1^ for CYN [50]. State-specific advisories do exist for ANA and STX in several states, but little information is available on BMAA, and therefore, there are no guidelines at this time [51]. In order to protect human and ecosystem health, more monitoring is essential for evaluating exposure risks, especially for these emerging substances.

For North Carolina (NC), only limited information is available on the presence of cyanotoxins. For instance, MCY was detected at low levels in 11 reservoirs across the Piedmont during summer 2002 [52], and in four lakes during 2011 and 2012 [53] (<0.8 µg L^−1^ MCY for all three studies). The EPA National Lakes Assessment 2007 also reported MCY and STX in NC waters, but CYN or ANA presence could not be confirmed [47]. While most previous records indicate a low MCY exposure risk based on WHO guidelines, concentrations of over 800 µg L^−1^, measured in Waterville Reservoir in October of 2007, are a reminder of how little is known about natural toxin ranges and their spatiotemporal dynamics in NC [54]. Most water treatment plants have procedures in place to eliminate toxins from drinking water [55,56], but the pervasiveness of cyanotoxins raises questions on chronic recreational exposure (e.g., swimming, boating, wading) [57,58] or the potential for food web poisoning via fish or shellfish consumption [10,57,59,60].

For this study, CyanoHAB dynamics were characterized in NC’s B. Everett Jordan Reservoir (henceforth, “Jordan Lake”), based on a six-year data set compiled through the North Carolina Division of Water Resources (NCDWR) Ambient Lakes Monitoring program. In addition to the continued collection of community structure data, the specific goal of this study was to test for the year-round presence of multiple cyanotoxins in an artificial reservoir that provides drinking water for nearly 300,000 people in locations such as Morrisville, Cary, and Apex. As the number of lakes and reservoirs that experience severe CyanoHAB blooms increases in the US and worldwide, newly developed approaches for measuring varying toxins are slow to be implemented in routine surveys. Although the presence of MCY was confirmed at multiple sites during summer 2002 (average 0.2 µg L^−1^) [47] within Jordan Lake, this is, to the best of our knowledge, the first comprehensive study to investigate five common cyanotoxins (2014–2016) using a combination of traditional and recently-developed tracking approaches. Cyanotoxin data were interpreted in relation to phytoplankton dynamics over multiple years (2011–2016) and in relation to pertinent environmental parameters throughout Jordan Lake.

## 2. Results

### 2.1. Phytoplankton Dynamics

**Figure 1:**
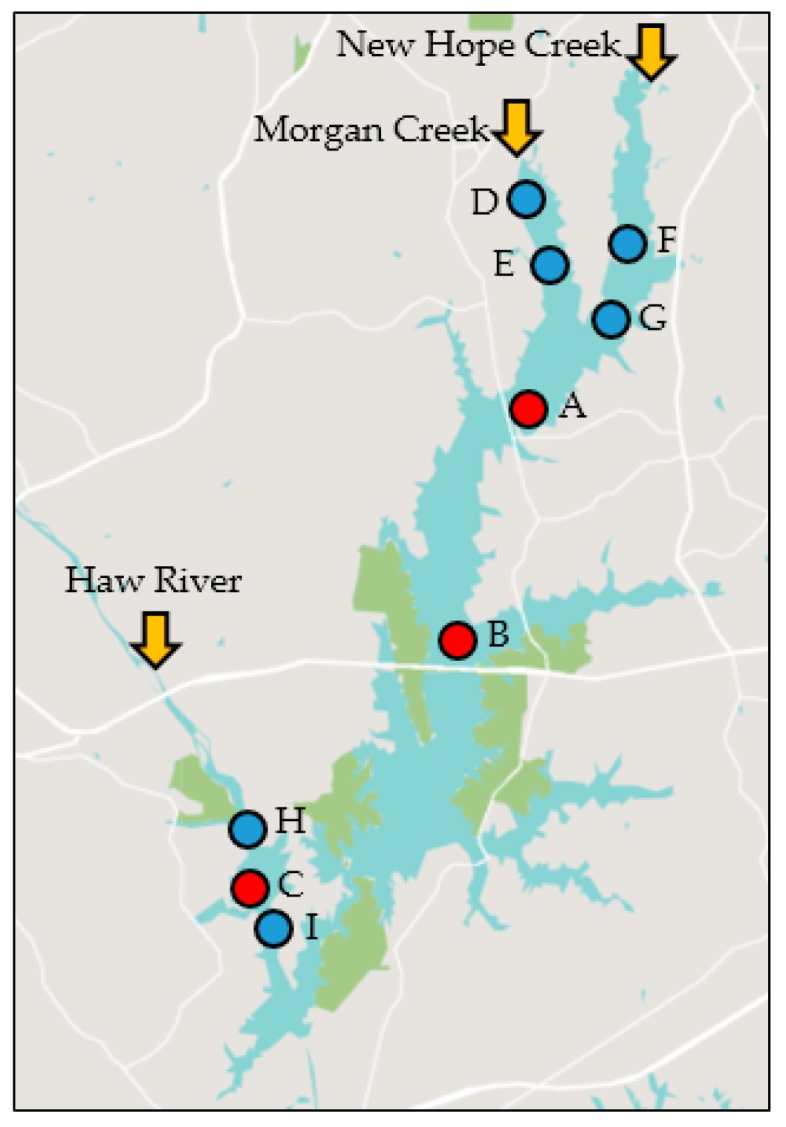
Map of Jordan Lake sampling sites. Biological, chemical and physical data were analyzed over a 6-year period (2011 to 2016) for sites A, B and C (red circles). Information over approximately 2 years (2014 to 2016) was available for an additional six sites (sites D through I, blue circles). Arrows indicate the three main rivers flowing into the lake. Map from snazzymaps.com.

**Figure 2:**
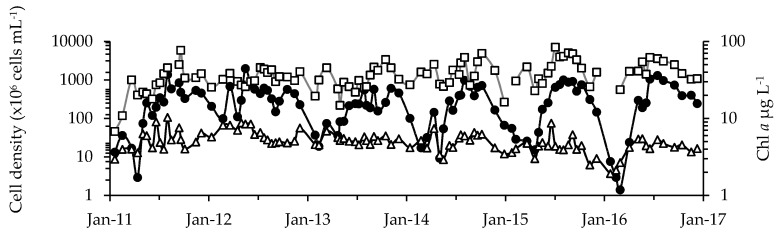
Average cell densities for cyanobacteria (black circles) and microeukaryote phytoplankton (white triangles) and for Chl *a* concentration (white squares). Before October 2014, averages were calculated for sites A through C. After October 2014, averages were calculated for all nine sites. Note: all axes are log-transformed.

**Figure 3:**
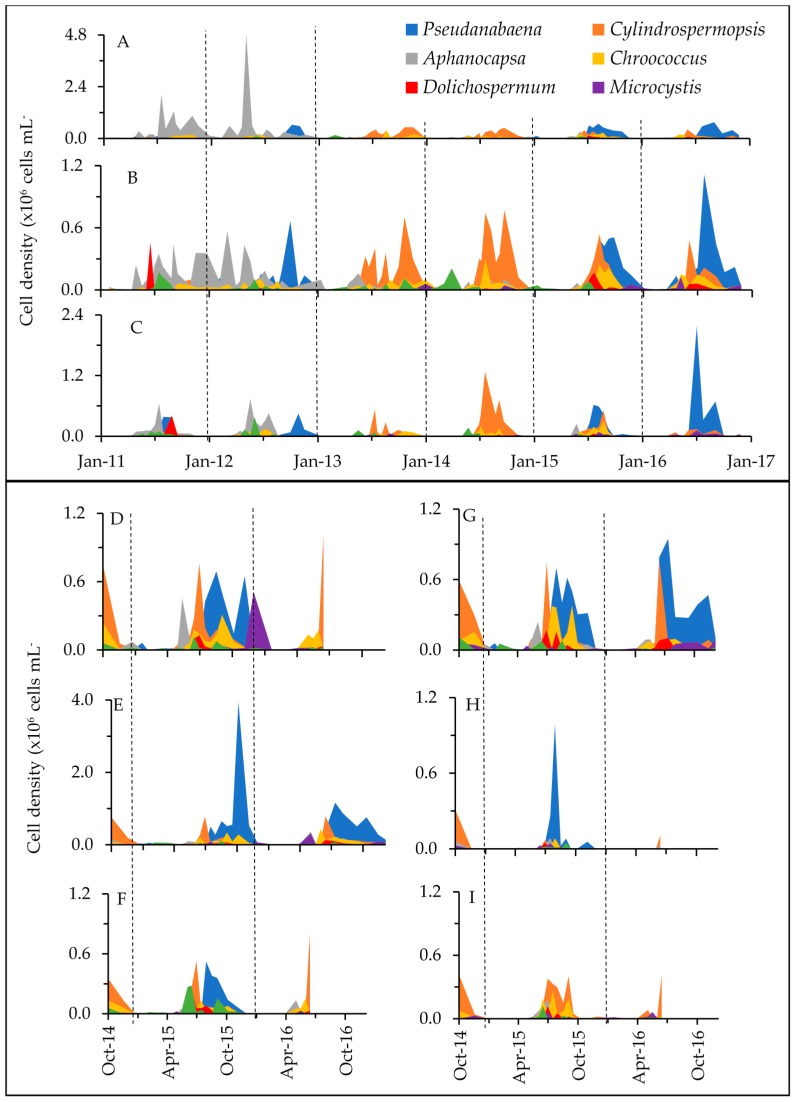
Cyanobacterial cell densities at each of the sampling locations (sites A through I are shown as panels A through I). Colors depict the six most abundant genera, and less abundant taxa are grouped as “Other”. Long-term sites A through C were sampled from January 2011 to December 2016. D, F, H and I were monitored from October 2014 to June 2016, while monitoring at E and G continued through December 2016. Vertical dashed lines separate years. Note, there are differing scales on the *y*-axes for A, C and E.

Overall, twelve cyanobacterial genera were identified with *Anabaenopsis*, *Aphanizomenon*, *Aphanocapsa*, *Aphanothece*, *Chroococcus*, *Cylindrospermopsis*, *Dolichospermum*, *Microcystis*, *Merismopedia*, *Planktolyngbya*, *Pseudanabaena*, and *Raphidiopsis* across the nine sampling sites (Figure 1, Table 1 and Table S1). In addition, 48 microeukaryote phytoplankton genera/species could be distinguished belonging to the diatoms, chlorophytes, chrysophytes, cryptophytes, euglenophytes, prymnesiophytes, and dinoflagellates (Table S1). Cyanobacteria dominated the phytoplankton assemblages based on cell counts (94% of total phytoplankton; range = 5% to 100%), while they constituted, on average, 39% (range ≤ 1% to 98%) to total phytoplankton biovolume. Cyanobacterial and microeukaryote phytoplankton abundance varied slightly with season, year and across the lake (*r* = 0.077 to 0.360 at *p* < 0.0003; three-way ANOSIM; Table S2). Peak densities were reached during summer (range = 4.3 × 10^3^ to 5.0 × 10^6^ and 180 to 3.2 × 10^5^, respectively) and fall months (250 to 4.3 × 10^6^ cells mL^−1^ and 180 to 1.1 × 10^5^, respectively; Figure 2 and Figure 3) and, in agreement with cell abundance, Chl *a* values reached their maximum during late summer/early fall, after a first initial increase typically during spring (overall range = 1 to 128 µg L^−1^; mean = 41 µg L^−1^; Figure 2).

**Figure 4:**
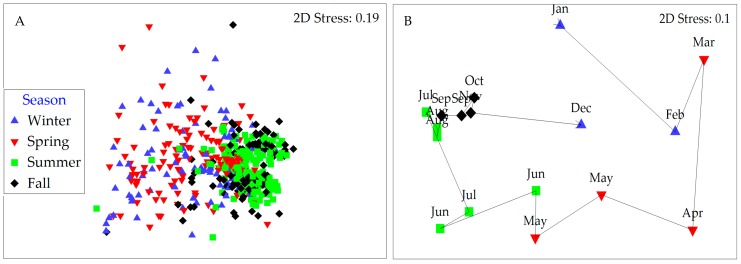
(**A**) MDS plot based on Bray–Curtis similarities for cyanobacterial communities as a non-metric multi-dimensional scaling (nMDS) plot by season (data from all years and stations combined). (**B**) Relative changes in cyanobacterial community composition shown along a month-to-month trajectory (site A in 2015). Stress values are reported in the top right corner of each plot.

Community structure based on Bray–Curtis similarities for both cyanobacteria and microeukaryote phytoplankton varied with season, site and year (*r* = 0.070 to 0.434; at *p* < 0.003; three-way ANOSIM; Table S3). For cyanobacteria, these similarities were highest during summer and fall (50% and 45% of the community were shared, respectively) compared to winter and spring (28% and 32%, respectively; Figure 4A). The less abundant microeukaryote assemblages consistently shared between 40% and 46% of their makeup within each of the seasons and throughout the year. Analyses of intra-annual community structure changes further revealed a recurrent pattern where the species composition followed a cyclic year-round pattern resulting in a “reset” of the assemblage by the onset of the following year (RELATE test; *ρ* = 0.022 to 0.639, *p* < 0.05). In total, 82% of the yearly datasets tested positive for cyclicity (shown for site A in 2015 in Figure 4B).

### 2.2. Physicochemical, Meteorological and Hydrological Parameters

**Figure 5:**
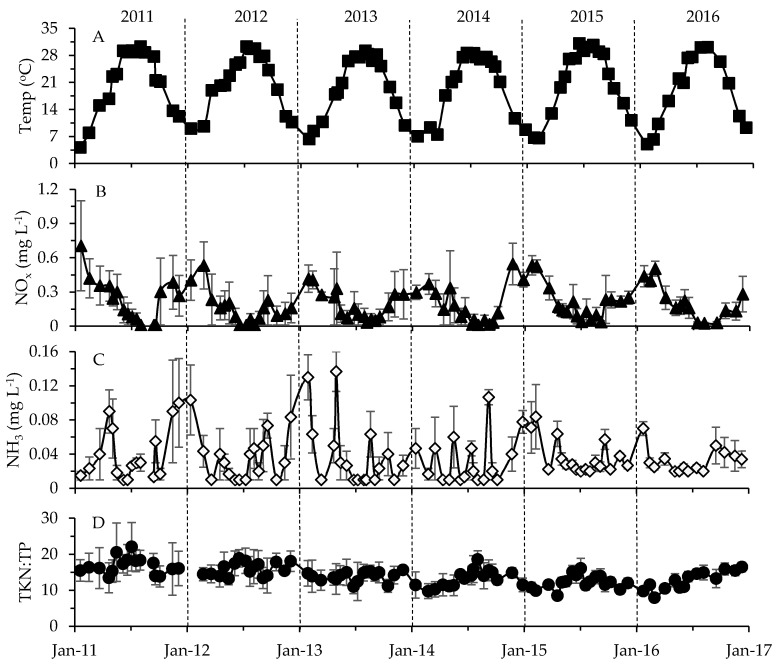
Changes in (**A**) temperature, (**B**) NO_x_, (**C**) NH_3_ concentration and (**D**) Total Kjeldahl nitrogen (TKN):TP ratio averaged for each sampling event. Standard error bars are included. Vertical dashed lines separate years.

Temperature, NO_x_ (nitrate plus nitrite) and dissolved oxygen (DO) concentrations in surface waters (surface to twice Secchi depth) showed the strongest seasonal changes (*r* = 0.146 to 0.614, *p* = 0.0001; one-way ANOSIM; Table S4), with temperature maxima in summer and NO_x_, DO and ammonia (NH_3_) levels higher during colder months (Figure 5). Total Kjeldahl nitrogen (TKN = particulate and dissolved organic N plus ammonia) and TKN:TP ratios slightly increased during summer months, while no consistent seasonal trends were observed over time for total phosphorous (TP = particulate and dissolved fractions; Table S4). NH_3_, DO and turbidity also varied somewhat between years (Table S4). All tested environmental parameters showed some spatial variability, except for temperature, NH_3_ and DO (*r* = 0.07 to 0.385, *p* = 0.0001; one-way ANOSIM; Table S4). Throughout the sampling period, surface waters at the sample location with the deepest water column depth of 12.2 m (site B) had some of the lowest NO_x_ and TP concentrations and the highest TKN:TP ratios (Table S5). NO_x_, TP and turbidity tended to be higher at some of the shallower sites (e.g., C, D, F and H in Figure 1, Table S5). Average TKN and TP concentrations ranged from 0.78 to 1.16 mg L^−1^ and 0.04 to 0.13 mg L^−1^, respectively (Table S5). Overall, mean surface DO levels (~0 to 3 m) ranged from 8.0 to 9.7 mg L^−1^ across the stations with individual measurements rarely falling beyond 4 mg L^−1^ (in <1% of measurements; Table S5).

**Figure 6:**
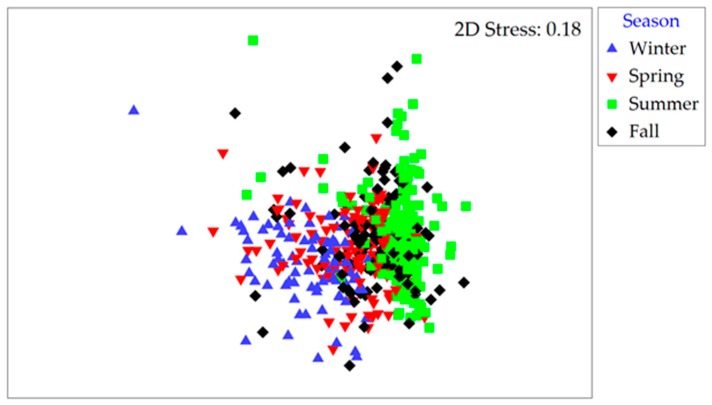
MDS plot showing Euclidian distances for environmental fingerprints by season. Parameters included in these analyses were temperature, NO_x_, NH_3_, TKN, TP and DO concentrations, TKN:TP ratios, pH levels and turbidity. The stress value is reported in the top right corner.

**Figure 7:**
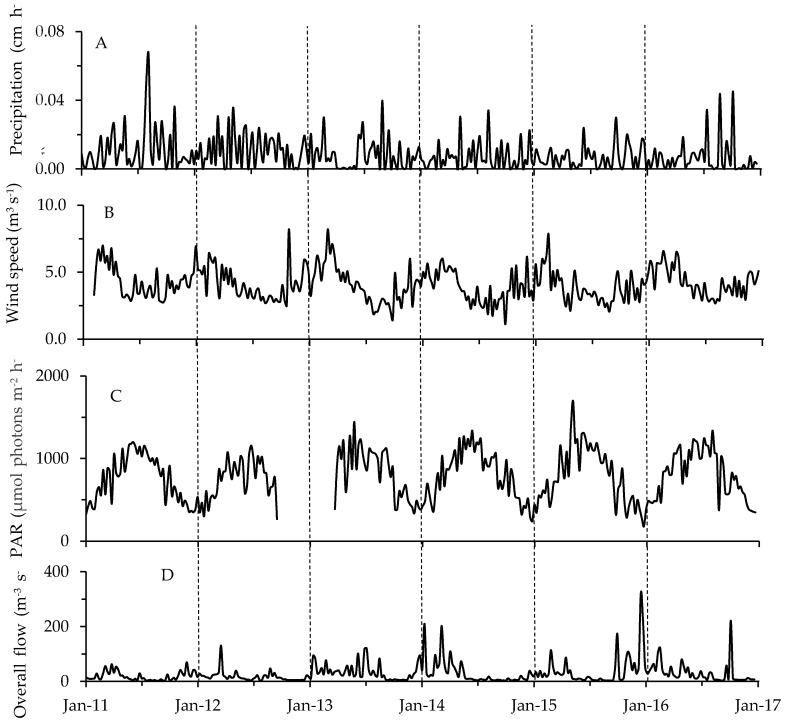
Weekly averages of meteorological and hydrological parameters: (**A**) precipitation; (**B**) wind speed; (**C**) PAR; (**D**) overall river flow. Vertical dashed lines separate years.

Combining all of the physicochemical data for each sampling event resulted in “environmental fingerprints” which varied with month, season, and site (average *r* = 0.16 to 0.348 at *p* = 0.0001; one-way ANOSIM; Figure 6). The most notable difference was seen when comparing physicochemical settings among seasons—conditions varied little throughout the summer (49% similar) compared to fall, winter and spring (<5% similar; Figure 6). Also available were single meteorological and hydrological values to characterize conditions across the entire lake during each sampling date (Figure 7, Table S6). Weekly precipitation averaged 0.02 cm h^−1^ with yearly maxima occurring throughout late spring to early fall (Figure 7A, Table S6). Wind speeds showed their maxima during the winter, while Photosynthetically Active Radiation (PAR) peaked in the summer (Figure 7B,C). Finally, overall river flow (Haw River, Morgan Creek and New Hope Creek combined) varied considerably from year to year with maxima typically occurring during spring or fall (Figure 7D). Haw River flow (mean = 27.79 m^3^ s^−1^) exceeded flows for Morgan Creek and New Hope Creek (0.12 and 0.14 m^3^ s^−1^, respectively).

### 2.3. Cyanotoxins

**Figure 8:**
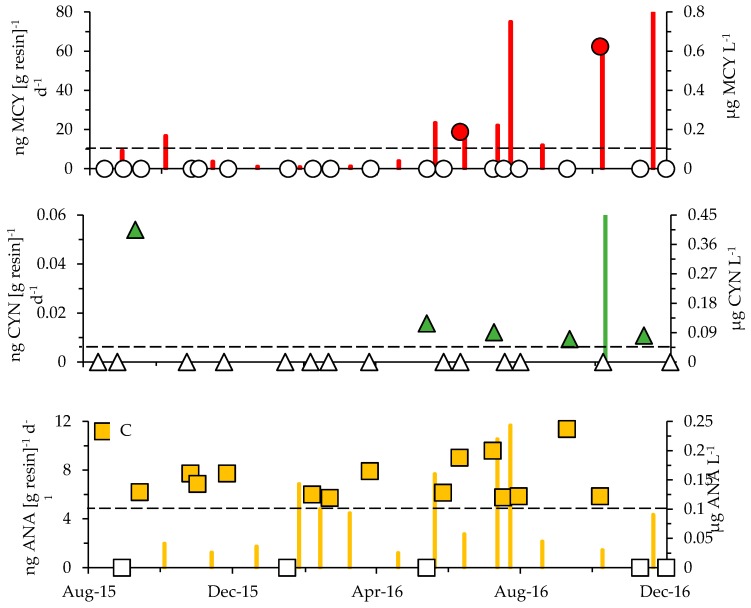
SPATT toxin values (columns) and toxin concentrations based on grab sampling (symbols) for (**A**) MCY, (**B**) CYN and (**C**) ANA for site E. SPATT toxin concentrations in ng toxin (g resin^−1^) d^−1^ are shown at the half-point of each deployment period. Grab samples are represented as µg toxin L^−1^ (filled symbols). Empty symbols along the *x*-axis indicate when toxin values fell below the LDL for each ELISA kit (LDLs shown as horizontal dashed lines originating from the secondary *y*-axes).

**Figure 9:**
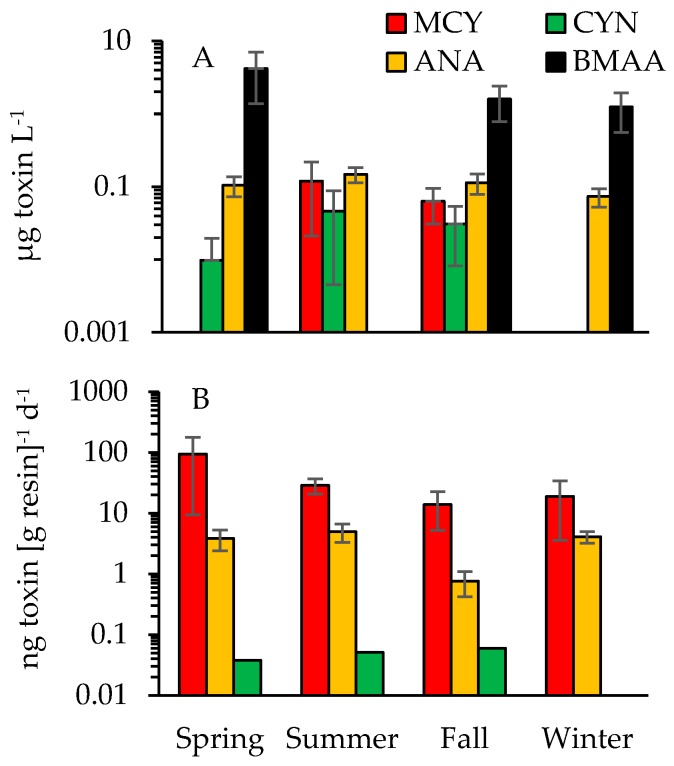
Seasonal averages for dissolved toxin concentrations based on (**A**) discrete sample analyses for sites A through G and (**B**) in-situ tracking (SPATTs) of MCY, ANA and CYN for sites E and G. Standard error bars are included whenever multiple samples tested positive. Note there are differing log-scales on the *y*-axes.

Four out of the five tested toxins were detected in Jordan Lake based on discrete (grab) samples collected between August 2015 and December 2016 at stations A through G (Figure 1). Dissolved MCY was confirmed in 10 out of 65 samples, ANA in 39 out of 69, CYN in six out of 63 and BMAA in nine out of 64 samples (Figure 8 and Figure 9, Table 2). STX presence could not be confirmed (*n* = 40; LDL (low detection limit) = 0.015).

In addition to the grab samples, in-situ toxin tracking was employed (Solid Phase Adsorption Toxin Tracking or SPATT) to confirm the presence of dissolved MCY, ANA and CYN at stations E and G (Table 2, Figure 8A). The combined sampling approaches revealed the occurrence of MCY at multiple sites (A, B and D through G) and throughout all seasons (Figure 9). SPATTs allowed for the confirmation of MCY in 92% of samples, but the toxin was only detected in 15% of discrete samples (Table 2). Dissolved CYN was measured at sites E and G during spring, summer and fall but not during winter (Figure 8 and Figure 9). CYN presence was indicated using both SPATT (13%) and grab (10%) sampling (Figure 8, Table 2). Similar to MCY, dissolved ANA was also found at multiple sites (A through G) and during all seasons (Figure 9). SPATTs confirmed ANA in 100% and grab sampling in 57% of tests across all sites (Table 2). Finally, dissolved BMAA was found at four sites (D through G) during fall, winter and spring (14% of samples; Figure 9, Table 3). All in all, multiple toxins were present at six out of seven sites and during 30 out of 69 sampling events. As stated earlier, to minimize loss of ANA and STX in lake water with a pH outside the range of 5 to 7, the addition of a diluent is recommended (Abraxis manual). Since samples prior to October 2016 were not treated with diluent and 96% of the lake water samples during that survey period measured above a pH of 7 (range = 5.3 to 9.5; mean = 7.8), both ANA and STX presence may have been underestimated in this study.

Due to a limited number of positives for CYN, STX and BMAA, statistical analyses to examine spatiotemporal trends were limited to dissolved MCY and ANA. MCY showed higher concentrations during summer and fall at sites E and G based on in-situ tracking (*r* = 0.194, *p* = 0.024; *n* = 24; one-way ANOSIM Table S7), but no trend was indicated based on grab sampling (*n* = 65). ANA concentrations did not vary significantly over time based on SPATTS data (*n* = 23), and while concentrations based on discrete sampling indicated some spatial variability (*r* = 0.094, *p* = 0.018; *n* = 69; one-way ANOSIM; Table S7), no consistent trend was apparent across the lake. For the most commonly detected toxins, MCY and ANA, concentrations were also examined for possible linkages to cyanobacterial composition shifts. For MCY, changes in concentration could be linked to shifts in genera—*Pseudanabaena*, *Merismopedia* and *Aphanothece*—while changes in *Raphidiopsis* spp. abundance linked to variance in ANA concentrations (Bio-Env [BEST] routine; *ρ* = 0.283 and 0.183, respectively, at *p* = 0.0001).

### 2.4. Linkages between Environmental Factors and Phytoplankton Dynamics

Correlation analyses indicated positive relationships for Chl *a*, cyanobacterial and microeukaryote density and biovolume (*r* = 0.314 to 0.851, *p* < 0.05; Table 3). Increases in dissolved MCY were correlated with increases in cyanobacterial density and biovolume as well as microeukaryote phytoplankton density and dissolved ANA (*r* = 0.270 to 0.408, *p* < 0.05). ANA showed a positive relationship with dissolved MCY, microphytoplankton biovolume and TKN:TP ratios (Table 3). Chl *a* as well as cyanobacterial and microeukaryote abundances correlated negatively with NH_3_ and NO_x_ but increased with TKN, temperature and pH (Table 3). Only the Chl *a* concentration was associated with increases in TP and turbidity (Table 3). There was no statistical significance when these analyses were conducted using average values for Chl *a*, cell densities and biovolumes across the lake (average for all stations) in regard to their relationships with meteorological and hydrological parameters (PAR, river flow, wind speed or precipitation).

Multiple regression analyses were performed and indicated that NH_3_, NO_x_, TKN and DO explained up to 68% of variance in Chl *a* (Table 4). NO_x_ and TKN, combined with pH and DO, were linked to 52% of variance in cyanobacterial densities and, without DO, explained up to 41% of variance in cyanobacterial biovolumes (Table 4). Only 13% and 19% of variance in microeukaryote phytoplankton densities and biovolume could be linked to a similar suite of physicochemical parameters (Table 4). Finally, community structure patterns (Bray–Curtis similarity matrices) were matched to varying combinations of the physicochemical, meteorological and hydrological variables (environmental fingerprints) using a BEST routine [61], and these trend analyses indicated that NO_x_ and temperature correlated most strongly with community structure patterns for cyanobacteria (*ρ* = 0.4 at *p* = 0.0001), and NO_x_, TKN and Morgan Creek flow data correlated with changed phytoplankton community structure for the microeukaryotes (*ρ* = 0.22 at *p* = 0.0001). No significant correlations were found when BEST routines were performed to match lake-wide community structure patterns to meteorological and hydrological variables.

## 3. Discussion

### 3.1. Cyanotoxins and Phytoplankton Dynamics in Jordan Lake 

CyanoHABs are a worldwide problem that has resulted in the development of WHO guidelines to assess risks (low, moderate and high) from MCY exposure based on toxin concentration, Chl *a* and cyanobacterial density [1,2,3,12]. However, applying these three metrics, a water body can be at risk based on one, but not all, of these criteria. For instance, for over 1100 lakes in the US, agreement for risk assessment based on all three parameters was only observed for 27% of the systems [47]. Given this discrepancy and the fact that most monitoring programs routinely measure Chl *a* and cyanobacterial density, but rarely employ approaches to measure toxins, complicates the tasks of water resource managers to protect designated lake uses and human health. Jordan Lake has been known for water quality issues due to eutrophication and recurrent CyanoHABs since its impoundment in the early 1980s. Despite its importance as a drinking water source for nearly 300,000 people and its recreational use by over a million visitors annually (NC Department of Natural and Cultural Resources [62], potential health risks from cyanotoxin presence had remained largely unexplored. Over a 2-year study period, from 2014 to 2016, a total of 36% of the examined samples from Jordan Lake tested positive for MCY but only one discrete sample (1.98 µg MCY L^−1^, site G on 21 June 2016) exceeded WHO guidelines for drinking water, with 1 µg MCY L^−1^, while values never reached those for EPA recreational guidelines of 4 µg L^−1^ [50,63]. The average concentration of dissolved MCY (0.06 µg L^−1^) was within the range of values observed in other NC freshwater systems (0.05 to 0.54 µg L^−1^) and across the US (BDL to 230 µg L^−1^) [47]. Notably, levels remained well below concentrations in CyanoHAB-prone systems, such as Lake Erie, where typical annual maxima peak at ~200 µg MCY L^−1^ and, in one instance, exceeded 1200 µg L^−1^ [64]. Following the aforementioned official WHO guidelines [47], Jordan Lake would be generally categorized as high risk based on its Chl *a* and cyanobacterial density; however, based on this study, only a low risk was observed for both MCY and CYN from 2014 to 2016. These findings further corroborate how critical toxin information is for the refinement of health risk metrics that directly inform lake-specific management decisions but also help shape national and international guidelines.

CyanoHABs may consist of multiple forms of toxins, but limited data is currently available on where and when toxins co-occur and under what environmental conditions [47,65,66,67]. To our knowledge, this study is unique in providing a year-round and multi-year record that allows the confirmation of the presence and co-occurrence of four cyanotoxins (dissolved MCY, CYN, ANA and BMAA) in a US freshwater body (Table 2 and Table 5). In contrast to this study, previous state-wide surveys tested a small number of samples (~seven or less) and these were typically collected during one season. Such limited temporal coverage is common and increases the probability of missing toxic events in any water body, due to the ephemeral nature of CyanoHABs. In NC, for instance, the detection of an unprecedented high MCY level of over 800 µg L^−1^ in Waterville Reservoir in 2007 raises questions on how well natural toxin ranges and spatiotemporal dynamics have been assessed based on traditional grab sampling and existing monitoring frequencies [54]. CYN had been tested for, but was not detected in, any major NC freshwater body [47,53]. ANA genes were found in six lakes (City Lake, Oak Hollow Lake, Randleman Reservoir, Lake Brandt, Lake Mackintosh, and Belews Lake) but the toxin itself was not confirmed [47,53]. Little to no information is currently available on BMAA presence or STX in NC lakes and reservoirs. US-wide, STX was confirmed in 7.7% of lakes during the National Lakes Assessment 2007, and this survey included one NC lake: Lake Rhodhiss (Table 5) [47]. Universally, more comprehensive datasets are needed to aid the development of risk thresholds for newly emerging cyanotoxins (e.g., no national guidelines currently exist for ANA or BMAA [51,68], to allow comparisons across freshwater systems and regions and to begin to inform epidemiological studies on the possible synergistic effects of multiple toxins.

A lack of information on co-occurring toxins typically goes hand-in-hand with limited data on year-round toxin dynamics. In this study, discrete toxin sampling that provided momentary snapshots of conditions was complemented by year-round in-situ tracking (SPATTs approach) at two sites in Jordan Lake, from 2014 to 2016. The advantages of employing SPATTs come from their higher sensitivity in detecting low toxin levels via a time-integrative signal, their use in freshwater to marine environments, the facilitation for testing multiple toxins, and their easy deployment and recovery [69]. A major limitation of using passive samplers, especially as a sole toxin detection approach, comes from the semi-quantitative nature of the data that can currently not be linked to regulatory limits and, hence, makes risk determination in systems difficult [69]. SPATT-based average MCY concentrations in Jordan Lake (36.2 ng (g resin)^−1^ d^−1^) fell within the ranges reported in several California studies (19.6–137.7 ng (g resin)^−1^ d^−1^) [70,71], and, similar to those reports, the SPATT method proved more sensitive for MCY detection compared to grab sampling. In Jordan Lake, MCY was present year-round, with 92% of the tested samples based on in-situ tracking compared to only 15% based on grab sampling (Table 2). This supports the effectiveness of in-situ tracking approaches in addressing emerging concerns in regard to the potential impacts of chronic or subacute exposure for wildlife and humans [72,73]. Using SPATTs for the detection of cyanotoxins other than MCY requires careful consideration of resin type [74]. As such, the hydrophobic HP-20 resin used for this study was thoroughly tested for its efficiency in detecting MCY but has not been fully evaluated for its efficiency in adsorbing other toxin types (e.g., CYN or ANA) [74]. For instance, only a total of three samples tested positive for CYN in this study (Figure 9B, Table 2). While both SPATTs and grab samples allowed for consistent detection of ANA, relative concentrations based on SPATTs were relatively low compared to MCY levels based on in-situ accumulation. This difference could have been a direct consequence of ANA being less prevalent throughout deployment periods, which would lower accumulation potential, but was likely also an artifact of the toxin not being efficiently adsorbed and/or retained during prolonged deployment [74,75]. An increasing number of studies have been conducted to test resins for the detection of algal toxins, to better evaluate the potential of in-situ passive samplers, to inform future health risk assessments and management decisions (review in [74]).

Phytoplankton assemblages in Jordan Lake were dominated by cyanobacteria (~94% based on cell density) with microeukaryote phytoplankton only rarely outnumbering the prokaryotes during non-bloom months. Changes in overall community structure followed consistent intra-annual patterns for both cyanobacteria and less abundant microphytoplankton. All of the six most abundant cyanobacterial taxa, identified via microscopy, were potential producers of single or multiple toxins, which included *Pseudanabaena* spp. (MCY), *Cylindrospermopsis raciborskii* (CYN, ANA, STX, BMAA), *Aphanocapsa delicatissima* and *A. pulchra* (MCY), *Chroococcus* spp. (MCY), several species of *Dolichospermum* (MCY, CYN, ANA, STX, BMAA) and *Microcystis aeruginosa* and *M. firma* (MCY, BMAA) [76,77]. Of these main genera, *Aphanocapsa*, *Cylindrospermopsis* and *Pseudanabaena* occurred in 2-year dominance shifts, a pattern that could not be linked to any of the physicochemical or hydrological factors tested in this study. Exploring the relationships between toxin presence and cyanobacterial community data indicated that relative changes in the abundance of *Pseudanabaena* spp., *Merismopedia punctata* and *Aphanothece saxicola* were linked to shifts in MCY, and *Raphidiopsis* spp. abundances were associated with changes in dissolved ANA [78,79,80,81]. Whether these taxa were truly responsible for toxin production remains unconfirmed and would have required further taxonomic resolution on the species and strain levels, since toxicity is not a genus-specific trait, nor is toxin production continuous. Combining field studies, such as this, with culture-based trials using isolates will allow us to verify taxonomic affiliations based on genomics, explore gene expression and tie findings to meta-omics profiles for natural cyanobacterial communities [28,53].

### 3.2. Environmental Factors in Relation to Phytoplankton and Toxin Dynamics

Jordan Lake has been consistently rated as eutrophic or hyper-eutrophic, and nutrient input from urban (26%) and agricultural (16%) land uses upstream (the remaining 58% are forested) serves as important stimulant for phytoplankton growth [82]. NO_x_ and NH_3_ concentrations were positively correlated with overall river flow in this study, and the availability of both nitrogen sources subsequently declined with increasing algal biomass, cell densities and total TKN. This overall shift in TKN was most likely attributed to the incorporation of N into algal biomass. A suite of environmental factors, including NO_x_, TKN, NH_3_, DO and pH, was linked to 68% of the variance in Chl *a* and 52% in cyanobacterial density (Table 4). In contrast, TKN and NO_x_ were associated with only 19% of the variance observed for microeukaryote phytoplankton, indicating that these main algal groups flourish under different environmental conditions. In agreement with studies elsewhere, changes in temperature, together with nutrient availability (i.e., NO_x_), were linked to shifts in overall cyanobacterial community structure [9,25,28,83]. Only a weak correlative relationship was observed between microphytoplankton composition and a combination of NO_x_, TKN, and river flow (Morgan Creek). Information on additional key environmental factors is needed to further characterize the significance of these potential forcing factors for microphytoplankton but also for cyanobacterial and toxin dynamics in Jordan Lake. For instance, urea has been reported to specifically stimulate cyanobacteria [16,84], and changes in the availability of both urea and inorganic P have been linked to increased abundances of toxic species [85,86]. Additionally, shifts in N:P ratios have been suggested to promote N-fixing cyanobacteria [17,87], a group also represented in Jordan Lake (i.e., genera *Cylindrospermopsis*, *Dolichospermum* and *Pseudanabaena*) [88,89,90]. Examining potential linkages for the two most commonly detected toxins, ANA and MCY, only revealed a positive correlation between TKN:TP ratios and dissolved ANA, giving some indication that P might have been less readily available relative to N. However, this remains speculative since TKN and TP estimates included varying dissolved and particulate fractions, and no separate information was obtained on the availability of dissolved P to further explore relationships between toxin and dissolved versus cell-bound nutrients. As cyanotoxin production may be tied to a complex array of environmental conditions, the collection of larger toxin datasets seems imperative for deciphering these linkages.

Finally, the role that river flow and water retention time play for CyanoHAB dynamics in Jordan Lake warrants closer examination in future studies. The lower arm of the lake, where river input is higher due to the Haw River (90% of water inflow into the lake), has estimated retention times of about 5 days, while they reportedly exceed 400 days in the upper arms (New Hope and Morgan Creeks) of Jordan Lake (NCDWR). Previous studies have shown that low flow conditions tend to result in larger blooms and shifts in species composition [22,24,91,92]. However, examining correlations between algal abundances and/or community structure among individual stations or regions (grouped stations adjacent to river outflows) did not yield significant differences. Moreover, and in contrast to previous studies that reported drought conditions favorable to bloom activity [23,52,93], no correlative relationships were seen between precipitation, algal abundances and community structure over the investigated study period.

### 3.3. Conclusions and Recommendations

This study is the first to show that cyanobacterial communities in Jordan Lake are linked with the recurrence of multiple cyanotoxins throughout the year. These findings fall in line with an increasing number of studies that have confirmed the ubiquitous nature of cyanotoxins, their simultaneous presence in varying environments and the need for further research to characterize the conditions that favor toxin production. The continued development and employment of highly sensitive toxin-tracking approaches (e.g., SPATTs), together with an expanding tool-kit for genomic and transcriptomic testing, will be essential for examining cause–effect relationships and providing the knowledge needed to predict the likelihood for toxin exposure via varying exposure pathways, be it to single or multiple agents. The presented study approach can inform efforts in similar water bodies where continued issues with eutrophication due to increasing population growth threaten water quality. The study’s findings serve as a baseline to better characterize CyanoHAB events in Jordan Lake and guide continued testing for selected toxins as part of routine water quality monitoring to protect the lake’s dedicated uses (i.e., drinking water and recreation).

## 4. Materials and Methods

### 4.1. Study Area and Data Collection

Jordan Lake is a 56 km^2^ artificial reservoir in central NC in Chatham County, constructed between 1967 and 1983 by the U.S. Army Corps of Engineers. The lake is filled by the Haw River in the south and Morgan Creek and New Hope Creek in the north (Figure 1). The Haw River is the largest of the three inflows and accounts for 70–90% of the total water volume entering the lake [94]. The reservoir has an average depth of 4.9 m and volume of 265 × 10^6^ m^3^ [52,95]. Sampling by NCDWR was conducted via small boats, as outlined in the DWR manual for standard operating procedures [96,97]. Briefly, Secchi depth was measured, and physical data (temperature, DO, and pH) were collected from the surface to depth at approximately 1 m intervals throughout the water column, using either Hydrolab (Hach Environmental, Loveland, CO, USA) or YSI (Yellow Springs Instrument Co., Yellow Springs, OH, USA) sondes. Grab samples for community structure and chemical analyses (NH_3_, NO_x_, TKN, TP and turbidity) were collected via a depth-integrated sampler from the surface to twice Secchi depth. Transport and processing for phytoplankton, nutrients, Chl *a* and turbidity followed standard protocols detailed in DWR’s standard operating manual [97]. Drought index measurements were obtained from DWR’s online Drought Monitor History database based on weekly drought averages for drought conditions by percent area for Chatham County (representative of the upper Cape Fear River watershed). Hourly meteorological parameters (wind speed, precipitation, and PAR) were obtained from the Reedy Creek Field Laboratory (State Climate Office of North Carolina), located approximately 27 km from Jordan Lake (35.807° N, 78.744° W). Daily hydrological data, obtained from the US Geological Survey (USGS), included stream discharge (flow) for Morgan Creek (near Chapel Hill, NC at 35.89333° N, 79.01972° W, site ID 02097517), New Hope Creek (near Blands, NC at 35.885° N, 78.96528° W, site ID 02097314) and Haw River (near Bynum, NC at 35.76528° N, 79.13583° W, site ID 02096960).

### 4.2. Phytoplankton Data

Phytoplankton community analyses were conducted microscopically by NCDWR using Leitz inverted microscopes and Utermöhl counting chambers [98,99]. Briefly, samples were preserved with Lugol’s solution (0.4% final concentration) upon collection, and a 5 mL subsample was settled for 24 h. Samples were analyzed within 14 days of collection. Samples were counted until 100 units (single cells, colonies or filaments, depending on the specific taxon) of the most dominant taxa were recorded. Taxonomic identification [100] was established to at least genus level. Biovolumes were calculated using cell densities (cells mL^−1^) multiplied by reference values [99].

### 4.3. Toxin Analyses

Discrete (grab) samples were collected at approximately 0.5 m depth using pre-cleaned (acid washed followed by three Milli-Q [MQ] water rinses) polyethylene terephthalate glycol (PETG) bottles and after pre-rinsing the bottles with lake water. The bottles were chilled on ice in a cooler for transport to the lab, where 50 mL aliquots were filtered through GF/F filters with a nominal 0.7 µm pore size (Whatman grade, GE Healthcare Life Sciences, Chicago, IL, USA). The filtrate was collected in glass scintillation vials for analysis of dissolved (extracellular) toxins and stored frozen at −20 °C until analysis using commercially available ELISAs (Abraxis Inc., Warminster, PA, USA; see details below). Samples were analyzed using a BioTek ELx800 Absorbance Microplate Reader (BioTek, Winooski, VT, USA). Dissolved samples for ANA and STX were pretreated with a diluent to prevent toxin loss, following the manufacturer’s guidelines (Abraxis), except for samples collected prior to October 2016. Toxin analyses were conducted for sites A through G from August 2015 through December 2016.

In addition to grab samples, Solid Phase Adsorption Toxin Tracking (SPATT) [71,101] units were used to determine in-situ toxin accumulation over approximately monthly intervals (average deployment time was 28 days). SPATTs were deployed at 2 sites at 0.5 m depth (Figure 1, sites E and G) from August 2015 to December 2016. Construction, deployment and extraction procedures for SPATTs followed previously published guidelines [101]. Briefly, 3 g of HP-20 resin (Sigma–Aldrich, St. Louis, MO, USA) was activated in 100% methanol for 30 min, then rinsed with three equivalent volumes of MQ-water and sonicated for 45 s at 50% amplitude with a sonic dismembrator (Fisher Scientific, Hampton, NH, USA, Model FB120). After sonication, activated bags were stored in chilled MQ water in the refrigerator until deployment [101]. Buoys with a weighted rope were deployed with a mesh bag containing two SPATTs attached at sites E and G. Retrieved units were kept out of direct sunlight, put on ice for transport to the lab and transferred into a −80 °C freezer within ~2 h of collection. The resin from the SPATT bags was extracted according to previously published protocols [70] with the following modifications: samples were vortexed before each of the three extractions and extracts two and three were combined for analysis, while extract one was run separately. All cyanotoxin analyses for SPATT extracts and dissolved samples were conducted using ELISAs (Abraxis Inc., Warminster, PA, USA): MCY-ADDA (Product #520011; sensitive to MCY-LR, -YR, -LF, -RR, LW, and nodularin; LDL = 0.10 µg L^−1^), CYN (Product #522011; sensitive to CYN and deoxy-CYN; LDL = 0.04 µg L^−1^), ANA (Product #520060; sensitive to anatoxin-a and homoanatoxin-a; LDL = 0.1 µg L^−1^), STX (Product #52255B; sensitive to STX and other paralytic shellfish poison [PSP] toxins; LDL = 0.015 µg L^−1^), and BMAA (Product #520040; sensitive to BMAA and other amino acids; limit of quantitation = 4 ng mL^−1^). Immediately prior to analysis, SPATT samples were diluted with the sample diluent provided with each ELISA kit to avoid methanol interference during assays. Final methanol concentrations were <5% for MCY, <20% for CYN and <2.5% for ANA (ELISA manuals and Abraxis recommendations). In addition, diluted SPATT extracts were centrifuged for 2 min at 13,000 rpm at room temperature (Eppendorf 5424 R Microcentrifuge) to remove any particulate matter. SPATT results were normalized as nanograms toxin per gram resin per day (ng toxin (g resin)^−1^ d^−1^). STX and BMAA were not analyzed using the SPATT approach due to adsorption bias when using HP-20 resin (see further details in the discussion) [74].

### 4.4. Chlorophyll Analyses

Chl *a* concentration (µg L^−1^) was determined using the EPA method 445.0 via fluorescence [102]. Briefly, 50–100 mL aliquots of lake water were concentrated onto 0.7 µm GF/F filters, extracted using acetone and measured fluorometrically (Turner Designs Model 10 Series fluorometer).

### 4.5. Statistical Analyses

Statistical analyses were performed using the PRIMER v7 [61] and STATISTICA 13 (TIBCO Software) statistical software packages. Community data (cell densities and biovolumes) were square-root transformed and compared based on Bray–Curtis similarity values, while physical environmental parameters (averaged over the upper water column from the surface to twice Secchi depth) were log-transformed, normalized (mean subtracted from each value and divided by the standard deviation) and compared after the computation of Euclidean distance resemblance matrices [61]. Three-way ANOSIM (analysis of similarity) tests were computed to examine temporal (seasonal [spring: March–May; summer: June–August; fall: September–November; winter: December–February] and yearly) as well as spatial trends (crossed design; 9999 permutations). This resulted in *r* values which represent a measure of distinction between groups. For instance, *r* values of 0 indicated that groups were similar, while an *r* value of 1, or close to 1, implied that groups were dissimilar. Similarity patterns over temporal or spatial scales were further illustrated using non-metric multidimensional scaling (nMDS) plots where more closely clustered data points represented higher similarity. Stress values were calculated for MDS plots to reflect the level of distortion that results from representing similarity rankings between multiple samples in a two-dimensional space. Generally, a stress value of <0.2 indicates an accurate representation of similarity rankings [61]. Additionally, one-way ANOSIM tests were conducted to examine whether toxin concentrations varied over time and location. Cyanobacteria and phototrophic microeukaryotes were analyzed separately and combined (total phytoplankton), and temporal or spatial differences were determined based on abundance data (cells mL^−1^) as well as biovolume (mm^3^ m^−3^). In contrast to the environmental data, phytoplankton data was square-root transformed and compared using Bray–Curtis similarity indices [61].

Chl *a*, cell densities, biovolumes and toxin concentrations were examined for their relationships with physical (temperature, DO and pH) and chemical (NH_3_, NO_x_, TKN, TP and turbidity) parameters using correlation and regression analyses (Pearson’s product-moment correlations, *r*; adjusted coefficient of determination, R^2^; STATISTICA 13, TIBCO Software). The same analyses were conducted using average values for Chl *a*, cell densities and biovolumes across the lake (all stations combined per sampling date) and related to meteorological and hydrological parameters (PAR, river flow, wind speed or precipitation). A BEST routine (PRIMER v7) was used to establish matches between similarities in site-specific physicochemical data (Euclidean distance-based matrix) and community structure information (Bray–Curtis similarity matrix) using Spearman’s rank correlations (rho, *ρ*) [61]. This routine was also repeated to link community data across the lake (all stations combined per sampling date) to meteorological and hydrological parameters. The BEST routine, unlike multiple regression analyses, cannot differentiate among positive or negative relationships, but identifies similarities between the two matrices. The RELATE test was used to test a cyclical model for an annual resetting of algal and microeukaryote assemblages at differing stations and in each of the 6 years [61].

## Figures and Tables

**Table 1:** Latitude (Lat) and longitude (Long) for the nine sampling sites across Jordan Lake. Sampling was conducted on a monthly basis with more frequent biweekly monitoring during months with higher bloom activity (May through September). Included are the Site ID and Division of Water Resources (DWR) site names. *n* = number of sampling time points per site. Depth describes the average water column depth.

Site ID	DWR ID	Lat (° N)	Long (° W)	From	To	*n*	Depth (m)
A	CPF086C	35.794	79.004	January 2011	December 2016	98	5.06
B	CPF087D	35.742	79.021	January 2011	December 2016	96	7.67
C	CPF055C	35.687	79.083	January 2011	December 2016	95	5.87
D	CPF086CUPS	35.837	79.001	October 2014	June 2016	26	1.47
E	CPF086C	35.825	78.998	October 2014	December 2016	34	2.92
F	CPF081A1B	35.836	78.976	October 2014	June 2016	25	1.82
G	CPF081A1C	35.815	78.983	October 2014	December 2016	32	3.16
H	CPF055C1	35.699	79.082	October 2014	June 2016	27	2.25
I	CPF055C6	35.682	79.078	October 2014	June 2016	27	8.50

**Table 2:** Percentage (%) of samples that tested positive for varying toxins using discrete sampling and the Solid Phase Adsorption Toxin Tracking (SPATTs) approach. Average concentrations (Ave) for dissolved (Diss) toxins are shown as µg L^−1^ (values below LDL were not included when calculating the average for each toxin), and for SPATT as ng toxin (g resin)^1^ d^−1^. *n* = number of samples tested. LDL = low detection limit of Enzyme-Linked Immunosorbent Assay (ELISA) detection method; BDL = below detection limit of ELISA test; MCY = microcystin; ANA = anatoxin-a; CYN = cylindrospermopsin; BMAA = β-*N*-methylamino-l-alanine; STX = saxitoxin.

Toxin	Sample Type	Ave	Range	Positive (%)	*n*	LDL
MCY	Diss	0.37	BDL—1.98	15	65	0.10
	SPATT	39.49	BDL—347.45	92	24	
ANA	Diss	0.2	BDL—0.68	57	69	0.10
	SPATT	3.97	0.31—13.28	100	23	
CYN	Diss	0.27	BDL—0.83	10	63	0.04
	SPATT	0.05	BDL—0.05	13	24	
BMAA	Diss	10.75	BDL—23.45	14	64	4.00
STX	Diss	BDL	BDL	0	40	0.015

**Table 3:** Results from correlation analyses. Pearson’s correlation coefficients (*r*) shown in bold are significant at *p* < 0.05. Cyano = cyanobacteria; Microphyto = microeukaryote phytoplankton; Diss = dissolved; DO = dissolved oxygen; Chl *a* = chlorophyll *a*.

	Chl *a* (µg L^−1^)	Cyano (Cells mL^−1^)	Cyano (mm^3^ m^−3^)	Microphyto(Cells mL^−1^)	Microphyto(mm^3^ m^−3^)	MCY (ng mL^−1^)	ANA (ng mL^−1^)
Cyano (cells mL^−1^)	**0.555**						
Cyano (mm^3^ m^−3^)	**0.515**	**0.851**					
Microphyto (cells mL^−1^)	**0.358**	**0.367**	**0.314**				
Microphyto (mm^3^ m^−3^)	**0.447**	**0.469**	**0.364**	**0.329**			
Diss MCY (ng mL^−1^)	0.101	**0.272**	**0.313**	**0.408**	0.055		
Diss ANA (ng mL^−1^)	−0.166	0.125	0.132	−0.008	**0.277**	**0.270**	
NH_3_ (mg L^−1^)	−**0.352**	−**0.257**	−**0.229**	−**0.154**	−**0.210**	−0.044	−0.135
NO_x_ (mg L^−1^)	−**0.540**	−**0.586**	−**0.499**	−**0.295**	−**0.349**	−0.102	−0.106
TKN (mg L^−1^)	**0.750**	**0.498**	**0.492**	**0.314**	**0.282**	0.147	−0.016
TP (mg L^−1^)	**0.217**	−0.022	0.061	0.049	−0.031	−0.023	−0.183
TKN:TP	**0.110**	**0.306**	**0.229**	0.089	**0.158**	0.200	**0.326**
Turbidity (NTU)	**0.164**	−**0.112**	0.002	0.068	−**0.105**	−0.075	−0.230
Temp (°C)	**0.322**	**0.524**	**0.470**	**0.175**	**0.238**	0.150	0.150
DO (mg L^−1^)	−0.018	−**0.309**	−**0.229**	−0.081	−**0.101**	−0.017	−0.125
pH	**0.441**	**0.458**	0.437	**0.200**	**0.231**	0.255	0.043

**Table 4:** Results from multiple regression analyses. Adj. R^2^ = adjusted coefficient of determination at *p* < 0.05. Individual t-statistics are listed in parenthesis. Turb = turbidity. Degrees of freedom for each regression = 441.

	Adj. R^2^	Parameter 1	Parameter 2	Parameter 3	Parameter 4	*n*
Chl *a*	0.680	NH_3_ (−8.61)	NO_x_ (−10.50)	TKN (19.99)	DO (5.66)	427
Cyano (cells mL^−1^)	0.521	NO_x_ (−9.10)	TKN (5.43)	DO (−5.56)	pH (8.62)	446
Cyano (mm^3^ m^−3^)	0.406	NO_x_ (−8.79)	TKN (6.31)	pH (6.19)		452
Microphyto (cells mL^−1^)	0.132	NO_x_ (−4.42)	TKN (5.05)			454
Microphyto (mm^3^ m^−3^)	0.189	NH_3_ (−3.12)	NO_x_ (−3.99)	TKN (5.34)	Turb (−3.71)	454

**Table 5:** Summary of reported cyanotoxins in North Carolina (NC) water bodies (based on discrete sampling). Average (Ave) concentrations denoted with an asterisk are approximated from published figures. BDL = below detection limit. ND = no data provided. *n* = replicates. Res = Reservoir. * = average value approximated from graph.

Location	Month/Year	Water Body	Toxin	Ave (µg L^−1^)	Range (µg L^−1^)	*n*	Method	Reference
Apex	August 2015–December 2016	Jordan Lake	MCY	0.06	BDL—1.98	65	ELISA	*This Study*
			CYN	0.02	BDL—0.83	63		
			ANA	0.11	BDL—0.68	69		
			BMAA	9.32	BDL—23.45	64		
Piedmont	June 2002–August 2002	Jordan Lake	MCY	0.20 *	ND	6	ELISA	[52]
		Kerr Scott Res	MCY	0.30 *	ND	6		
		Tuckertown Res	MCY	0.12 *	ND	6		
		Oak Hollow Lake	MCY	0.10 *	ND	6		
		Falls Lake	MCY	0.22 *	ND	6		
		Narrows Res	MCY	0.15 *	ND	6		
		Lake Rhodhiss	MCY	0.25 *	ND	6		
		Lake Michie	MCY	0.15 *	ND	6		
		High Rock Lake	MCY	0.05 *	ND	6		
		Lake Tillery	MCY	0.35 *	ND	6		
		High Point Lake	MCY	0.12 *	ND	6		
Piedmont	June 2011–September 2012	City Lake	MCY	0.22	BDL—0.31	6	ELISA	[53]
		Oak Hollow Lake	MCY	0.21	BDL—0.26	4		
		Randleman Res	MCY	0.17	BDL—0.18	7		
		Lake Mackintosh	MCY	0.17	BDL—0.17	5		
Waterville	October 2007	Waterville Res	MCY	824.3	ND	3	LC-MS	[54]
Statewide	June 2007–July 2007	Lake Lee	MCY	0.21	0.17—0.24	2	ELISA	[47]
		Lake Rhodhiss	MCY	0.14	BDL—0.14	2		
			STX	0.03	BDL—0.03	2		
		Lake Orange	MCY	0.54	0.54	1		
		Lake Fisher	MCY	0.17	0.147	1		
		High Rock Lake	MCY	0.52	0.52	1		
		Lake Townsend	MCY	0.16	0.16	1		
		Falls Lake	MCY	0.28	0.28	1		
		Lake Hickory	MCY	0.16	0.16	1		
		Beaverdam Lake	MCY	0.23	0.23	1		
		Graham-Mebane Lake	MCY	0.11	0.11	1		

## Supplementary Materials

The following are available online at http://www.mdpi.com/2072-6651/10/2/92/s1, Table S1: Most abundant cyanobacterial and microeukaryote phytoplankton groups, identified to genus or, where possible, species level using microscopy, Table S2: Results of three-way ANOSIM tests comparing cyanobacteria, microeukaryote phytoplankton and total phytoplankton abundances across season, year and site (crossed design), Table S3: Results of three-way ANOSIM tests comparing cyanobacteria, microeukaryote phytoplankton and total phytoplankton community structure across season, year and site (crossed design), Table S4: Results of one-way ANOSIM comparing environmental, meteorological and hydrological parameters across month, season, year and site, Table S5: Minimum (Min), maximum (Max) and average (Ave) values for parameters at each site, Table S6: Minimum (Min), maximum (Max) and average (Ave) values for meteorological and hydrological parameters, Table S7: Results of one-way ANOSIM comparing MCY and ANA concentrations based on SPATTs data and ANA levels based on grab sampling across month, season, year and site.
