# Supplementary material for: Algal Blooms and Cyanotoxins in Jordan Lake, North Carolina
Source: Toxins (Basel). 2018 Feb 24;10(2):92. doi: 10.3390/toxins10020092 (PMC5848192; doi:10.3390/toxins10020092)
Supplement: Supplementary file 1 [file toxins-10-00092-s001.docx]

Supplementary Materials: Algal Blooms and Cyanotoxins in Jordan Lake, North Carolina

Daniel Wiltsie, Astrid Schnetzer, Jason Green, Mark Vander Borgh and Elizabeth Fensin

**Table S1.** Most abundant cyanobacterial and microeukaryote phytoplankton groups, identified to genus or, where possible, species level using microscopy.

| **Group** | **Genus** | **Species** |
| --- | --- | --- |
| Cyanobacteria | *Anabaenopsis* | *circularis* |
|  | *Aphanizomenon* | spp. |
|  | *Aphanocapsa* | *delicatissima* |
|  |  | *pulchra* |
|  | *Aphanothece* | *saxicola* |
|  | *Chroococcus* | spp. |
|  | *Cylindrospermopsis* | *raciborskii* |
|  | *Dolichospermum* | *circinale* |
|  |  | *planctonicum* |
|  |  | *spiroides* |
|  |  | spp. |
|  | *Merismopedia* | *punctata* |
|  | *Microcystis* | *aeruginosa* |
|  |  | *firma* |
|  | *Planktolyngbya* | *undulata* |
|  | *Pseudanabaena* | spp. |
|  | *Raphidiopsis* | spp. |
| Diatoms | *Achnanthidium* | spp. |
|  | *Aulacoseira* | spp. |
|  | *Navicula* | spp. |
|  | *Synedra* | spp. |
|  | Unknown centric | spp. |
| Chlorophytes | *Ankistrodesmus* | spiriliformis |
|  | *Ankistrodesmus* | *falcatus* |
|  | *Coelastrum* | spp. |
|  | *Crucigenia* | *crucifera* |
|  | *Dictyosphaerium* | *pulchellum* |
|  | *Kirchneriella* | spp. |
|  | *Scenedesmus* | *quadricauda* |
|  |  | *bicaudatus* |
|  | *Tetrastrum* | *heterocanthum* |
| Chrysophytes | *Ochromonas* | spp. |
| Cryptophytes | *Cryptomonas* | spp. |
|  | *Komma* | *caudata* |
| Euglenophytes | *Trachelomonas* | spp. |
| Prymnesiophytes | *Chrysochromulina* | spp. |

**Table S2.** Results of three-way ANOSIM tests comparing cyanobacteria, microeukaryote phytoplankton and total phytoplankton abundances across season, year and site (crossed design). All global tests were run at 9999 permutations. R = sample statistic. P = significance level.

|  | Season | | Year | | Site | |
| --- | --- | --- | --- | --- | --- | --- |
|  | R | P | R | P | R | P |
| Cyanobacteria | 0.36 | 0.0001 | 0.091 | 0.0001 | 0.082 | 0.0003 |
| Microphytoplankton | 0.077 | 0.0001 | 0.102 | 0.0001 | 0.109 | 0.0001 |
| Total phytoplankton | 0.33 | 0.0001 | 0.102 | 0.0001 | 0.105 | 0.0001 |

**Table S3.** Results of three-way ANOSIM tests comparing cyanobacteria, microeukaryote phytoplankton and total phytoplankton community structure across season, year and site (crossed design). All global tests were run at 9999 permutations. R = sample statistic. P = significance level.

|  | Season | | Year | | Site | |
| --- | --- | --- | --- | --- | --- | --- |
|  | R | P | R | P | R | P |
| Cyanobacteria | 0.434 | 0.0001 | 0.3 | 0.0001 | 0.07 | 0.0003 |
| Microphytoplankton | 0.26 | 0.0001 | 0.333 | 0.0001 | 0.146 | 0.0001 |
| Total phytoplankton | 0.477 | 0.0001 | 0.361 | 0.0001 | 0.102 | 0.0001 |

**Table S4.** Results of one-way ANOSIM comparing environmental, meteorological and hydrological parameters across month, season, year and site. All global tests were run at 9999 permutations. R = sample statistic. NS = not significant at P < 0.05.

|  | **Month** | | **Season** | | **Year** | | **Site** | |
| --- | --- | --- | --- | --- | --- | --- | --- | --- |
|  | R | P | R | P | R | P | R | P |
| Temperature | 0.702 | 0.0001 | 0.614 | 0.0001 |  | NS |  | NS |
| Chl *a* |  | NS |  | NS |  | NS | 0.133 | 0.0001 |
| NO_x_ | 0.185 | 0.0001 | 0.194 | 0.0001 |  | NS | 0.215 | 0.0001 |
| NH_3_ | 0.086 | 0.0001 | 0.091 | 0.0001 | 0.183 | 0.0001 |  | NS |
| TKN | 0.022 | 0.018 | 0.015 | 0.042 |  | NS | 0.21 | 0.0001 |
| TP |  | NS |  | NS |  | NS | 0.385 | 0.0001 |
| TKN:TP |  | NS | 0.025 | 0.003 |  | NS | 0.335 | 0.0001 |
| DO | 0.197 | 0.0001 | 0.146 | 0.0001 | 0.031 | 0.015 |  | NS |
| pH | 0.103 | 0.0001 | 0.103 | 0.0001 |  | NS | 0.07 | 0.0001 |
| Turbidity | 0.021 | 0.012 | 0.038 | 0.0001 | 0.028 | 0.013 | 0.346 | 0.0001 |
| Precipitation | 0.054 | 0.0002 |  | NS | 0.044 | 0.011 |  |  |
| Wind speed | 0.154 | 0.0001 | 0.066 | 0.0001 | 0.118 | 0.0001 |  |  |
| PAR | 0.333 | 0.0001 | 0.253 | 0.0001 |  | NS |  |  |
| Morgan Creek flow | 0.221 | 0.0001 | 0.188 | 0.0001 | 0.094 | 0.0001 |  |  |
| New Hope Creek flow | 0.266 | 0.0001 | 0.193 | 0.0001 | 0.054 | 0.0003 |  |  |
| Haw River flow | 0.249 | 0.0001 | 0.201 | 0.0001 | 0.038 | 0.0006 |  |  |

**Table S5.** Minimum (Min), maximum (max) and average (Ave) values for parameters at each site. Averages for sites A, B and C were computed over 6 years; sites E and G over 2 years; and sites D, F, H and I over 1.5 years.

| **Site** |  | **Temp (^o^C)** | **Chl *a* (µg L^-1^)** | **NO_x_ (mg L^-1^)** | **NH_3_ (mg L^-1^)** | **TKN (mg L^-1^)** | **TP (mg L^-1^)** | **TKN:TP** | **DO (mg L^-1^)** | **pH** | **Turbidity (NTU)** | ***n*** |
| --- | --- | --- | --- | --- | --- | --- | --- | --- | --- | --- | --- | --- |
| A | Min | 4.37 | 9.00 | 0.01 | 0.01 | 0.70 | 0.04 | 7.69 | 3.23 | 5.33 | 6.50 | 98 |
|  | Max | 30.78 | 99.00 | 0.41 | 0.18 | 1.50 | 0.13 | 21.75 | 13.33 | 9.17 | 35.00 |  |
|  | Ave | 20.66 | 47.21 | 0.07 | 0.04 | 0.98 | 0.08 | 13.39 | 8.02 | 7.69 | 14.24 |  |
| B | Min | 6.26 | 7.10 | 0.01 | 0.01 | 0.58 | 0.00 | 12.67 | 4.60 | 6.57 | 3.40 | 96 |
|  | Max | 30.53 | 57.00 | 0.41 | 0.20 | 1.10 | 0.06 | 36.67 | 12.83 | 8.90 | 17.00 |  |
|  | Ave | 20.53 | 28.57 | 0.09 | 0.04 | 0.78 | 0.04 | 20.60 | 8.00 | 7.60 | 7.05 |  |
| C | Min | 4.04 | 4.60 | 0.01 | 0.01 | 0.50 | 0.04 | 5.08 | 4.10 | 6.77 | 4.30 | 95 |
|  | Max | 31.45 | 72.00 | 1.10 | 0.18 | 1.10 | 0.16 | 20.5 | 13.46 | 9.43 | 60.00 |  |
|  | Ave | 21.56 | 31.26 | 0.37 | 0.03 | 0.82 | 0.08 | 11.46 | 9.07 | 7.97 | 12.78 |  |
| D | Min | 5.65 | 21.00 | 0.01 | 0.01 | 0.72 | 0.06 | 7.50 | 5.59 | 5.47 | 11.00 | 26 |
|  | Max | 32.45 | 116.00 | 1.40 | 0.06 | 1.50 | 0.16 | 16.67 | 13.12 | 9.52 | 70.00 |  |
|  | Ave | 19.97 | 58.91 | 0.24 | 0.02 | 1.04 | 0.10 | 10.89 | 9.69 | 8.09 | 30.48 |  |
| E | Min | 5.29 | 20.00 | 0.01 | 0.01 | 0.68 | 0.05 | 7.86 | 4.97 | 5.47 | 7.90 | 34 |
|  | Max | 31.37 | 116.00 | 0.59 | 0.08 | 1.40 | 0.14 | 20.00 | 12.82 | 9.40 | 40.00 |  |
|  | Ave | 20.03 | 59.84 | 0.11 | 0.02 | 1.02 | 0.08 | 12.59 | 9.43 | 8.05 | 19.14 |  |
| F | Min | 4.95 | 9.10 | 0.02 | 0.01 | 0.63 | 0.05 | 6.54 | 4.67 | 5.43 | 14.00 | 25 |
|  | Max | 31.75 | 128.00 | 0.60 | 0.14 | 1.80 | 0.24 | 30.00 | 13.20 | 9.30 | 80.00 |  |
|  | Ave | 19.85 | 76.00 | 0.14 | 0.03 | 1.16 | 0.13 | 9.43 | 9.32 | 8.05 | 37.81 |  |
| G | Min | 4.65 | 23.00 | 0.01 | 0.01 | 0.71 | 0.05 | 8.57 | 4.61 | 5.57 | 7.90 | 32 |
|  | Max | 31.25 | 120.00 | 0.48 | 0.08 | 1.40 | 0.14 | 18.80 | 12.52 | 9.07 | 45.00 |  |
|  | Ave | 19.88 | 62.30 | 0.09 | 0.02 | 1.03 | 0.09 | 12.09 | 9.17 | 7.90 | 21.77 |  |
| H | Min | 4.24 | 1.00 | 0.09 | 0.01 | 0.45 | 0.05 | 5.14 | 6.10 | 6.91 | 5.00 | 27 |
|  | Max | 30.46 | 59.00 | 1.50 | 0.32 | 1.30 | 0.29 | 13.20 | 13.54 | 8.70 | 180.00 |  |
|  | Ave | 18.80 | 17.92 | 0.64 | 0.05 | 0.83 | 0.12 | 7.35 | 9.58 | 7.78 | 26.26 |  |
| I | Min | 4.54 | 6.90 | 0.02 | 0.01 | 0.50 | 0.05 | 5.56 | 5.47 | 7.00 | 5.00 | 27 |
|  | Max | 31.14 | 67.00 | 0.83 | 0.25 | 1.10 | 0.22 | 18.20 | 13.19 | 9.31 | 160.00 |  |
|  | Ave | 19.38 | 30.46 | 0.36 | 0.05 | 0.82 | 0.08 | 11.58 | 9.59 | 7.93 | 19.59 |  |
|  | Ave | 20.07 | 45.83 | 0.23 | 0.03 | 0.94 | 0.09 | 12.15 | 9.10 | 7.90 | 21.01 |  |
|  | Std dev | 0.79 | 19.61 | 0.19 | 0.01 | 0.13 | 0.03 | 3.64 | 0.65 | 0.65 | 9.45 |  |

**Table S6.** Minimum (Min), maximum (Max) and average (Ave) values for meteorological and hydrological parameters. Weekly precipitation, wind speed and PAR values were obtained from the Reedy Creek Field Laboratory, State Climate Office of North Carolina. Daily averaged river flows were provided by USGS.

| **Parameter** | **Min** | **Max** | **Ave** |
| --- | --- | --- | --- |
| Precipitation (cm hr^-1^) | 0.00 | 0.17 | 0.02 |
| Wind speed (m s^-1^) | 0.50 | 3.67 | 1.82 |
| PAR (µmol photons m^-2^ hr^-1^) | 180 | 1699 | 788 |
| Morgan Creek flow (m^3^ s^-1^) | 0.10 | 0.21 | 0.12 |
| New Hope Creek flow (m^3^ s^-1^) | 0.09 | 0.29 | 0.14 |
| Haw River flow (m^3^ s^-1^) | 1.5 | 275.5 | 27.8 |

**Table S7.** Results of one-way ANOSIM comparing toxin MCY and ANA concentrations based on SPATTs data and ANA levels also on grab sampling across month, season, year and site. All global tests were run at 9999 permutations. R = sample statistic. P = significance level.

|  | **Month** | | **Season** | | **Year** | | **Site** | |
| --- | --- | --- | --- | --- | --- | --- | --- | --- |
|  | R | P | R | P | R | P | R | P |
| MCY SPATT |  | NS | 0.194 | 0.024 |  | NS |  | NS |
| ANA SPATT |  | NS |  | NS |  | NS |  | NS |
| ANA dissolved |  | NS |  | NS |  | NS | 0.094 | 0.018 |
